# Circular RNAs 0064286 and 0000475: Potential Diagnostic Biomarkers in Hepatocellular Carcinoma

**DOI:** 10.31557/APJCP.2021.22.9.3039

**Published:** 2021-09

**Authors:** Fathia Zaky El Sharkawi, M. Samy Awad, Waleed Elagawy, H.A. Al-Sawaf, Heba Taha

**Affiliations:** 1 *Department of Biochemistry and Molecular Biology, Faculty of Pharmacy, Helwan University 11795, Ain Helwan, Cairo, Egypt. *; 2 *Department of Biochemistry, Faculty of Pharmacy, Egyptian Russian University, Egypt, Badr, Cairo-Suez Road 11829, Cairo, Egypt. *; 3 *Department of Tropical Medicine, Faculty of Medicine, Portsaid University, Egypt. *

**Keywords:** Circ RNA, HCC, metastasis diagnostic biomarkers

## Abstract

**Objective:**

Hepatocellular carcinoma (HCC) is considered the highest recorded malignancy in Egypt. The shortage of appropriate biomarkers for early detection often results in the late diagnosis of the HCC. Circular RNAs (CircRNAs) are presented as long stranded non-coding RNA that combine covalently to make a sealed circular form which make them very stable. CircRNAs are known to have interpretative role in cancer development and metastasis.

**Aim:**

To examine the dysregulation of two new CircRNAs obtained from Circbase database (hsa_circ_0064286 and hsa_circ_0000475) in the serum of HCC patients as predictable diagnostic biomarkers of HCC and their correlation with some liver biochemical parameters.

**Methods:**

Sixty clinically diagnosed HCC Egyptian patients and 25 healthy volunteers were enrolled in the study. Expression levels of the selected CircRNAs was evaluated in subjects’ serum. Moreover, correlation with liver biochemical parameters, sensitivity, and specificity of studied CircRNAs were estimated.

**Results:**

Both circular RNAs were significantly down regulated in HCC patients, which was negatively correlated with ALP, ALT, AST, AFP, and bilirubin levels. Circ_0064286 showed more sensitivity and specificity (88.3% and 96%, respectively).

**Conclusion:**

As far as we know, this is the first study that shed light on the expression levels of both circRNAs in Egyptian HCC patients. They may serve as potential biomarkers for HCC diagnosis. Moreover, those circRNAs draw attention as therapeutic targets for HCC through targeting their sponge miRNAs.

## Introduction

Hepatocellular carcinoma (HCC) is characterized by progressive genetic mutations resulting in altering the phenotype of the hepatocytes (Bruix et al., 2014). HCC ranks sixth in cancer prevalence, while in terms of mortality rate, it is the fourth worldwide (Bray et al., 2018).

In Egypt, hepatocellular carcinoma is considered the greatest recorded malignancy (Bray et al., 2018). Hepatitis viral infection as hepatitis B virus and hepatitis C virus, non – alcoholic fatty liver (NAFLD) and cirrhosis reflect the main causes of Egyptian rising incidence of HCC (Tang et al., 2018).

Imaging techniques shows a vital role in HCC diagnosis, multiphase computerized tomography (CT) or screening by magnetic resonance imagining (MRI) is acclaimed for initial diagnostic testing (Marrero et al., 2018). Also blood biomarkers would be extremely important for early diagnosis, prognosis and response to therapy in HCC patients. For many years, the most widely used blood biomarker was alpha-fetoprotein (AFP), however AFP has several limitations as lower sensitivity and weak specificity, making its use not satisfactory enough in the diagnosis of HCC (Chen et al., 2003). Therefore, there is an imperative necessity to develop new diagnostic biomarkers with higher sensitivity and specificity.

Circular RNAs (CircRNAs) are presented as one long stranded non coding RNA which combine covalently to make a sealed circular form (Lei et al., 2020), so they do not have 5’ or 3’ ends and are developed from a vast group of genomic fragments (Memczak et al., 2013; Miao et al., 2019). In vivo, it was shown that circular RNAs have high stability and efficient versatility. Micro RNA sponging is considered the main function of circular RNAs in many process (Skrzypek and Majka, 2020). On the other hand, Circular RNA possess the ability to regulate both gene transcription and expression (Lu, 2020) and interact with cell cycle proteins (Du et al., 2016; Yang et al., 2020). 

It has been shown that high quantities of stably expressed circular RNAs were present in the body fluids of homo sapiens, as plasma , sera and saliva, which grants circRNAs as perfect competitors biomarkers for cancer (Cui et al., 2018). Strong relationship among circular RNAs and a diversity of cancers were published including glioma, ovarian cancer, gastric cancer, liver cancer, esophageal cancer, and colon cancer (Ahmed et al., 2016; Dou et al., 2016; Qin et al., 2016; Xia et al., 2016; Yang et al., 2016; Chen et al., 2017).

The current study aimed to analyze the expression levels of two novel circular RNAs; hsa_circ_0064286 and hsa_circ_0000475 in the sera of Egyptian HCC patients and their correlation with the classical biochemical markers of liver disease. 

## Materials and Methods

*Subjects and sample collection*

Sixty clinically diagnosed hepatocellular carcinoma patients were enrolled from both inpatient and outpatient clinics of the national hepatology and tropical medicine research institute (NHTMRI), Cairo, Egypt. In addition to twenty-five healthy volunteers as controls with matched age, sex and ethnicity were included in the study, during the period from December 2017 to August 2018. 

The following data: child-Pugh classification, tumor number and size were obtained from patients’ files.

The study complied with Declaration of Helsinki and all patients agreed to participate in the study and gave written informed consent. The Human Research Ethics Committee of Helwan University approved all aspects of this study with approval number (04H2020).

Venous blood samples were drawn from each subject for serum separation and kept at -80°C till analysis; serum was separated for determination of hsa_circ_0064286, hsa_circ_0000475 and liver biochemical parameters [AFP, alkaline phosphatases (ALP), alanine transaminases (ALT), bilirubin, aspartate transaminase (AST), and albumin].

The exclusion criteria of HCC patients were; no previous treatment (chemotherapy or radiation) and no previous history of cardiovascular dieses, diabetes or any form of other malignancies.

*Selection of circular RNAs from data bases*

Circbase database was used to detect the circular RNAs which may be related to HCC, among circular RNAs, hsa_circ_0064286 and hsa_circ_0000475 (Alias: hsa_circ_001053) were selected for the present work. Then Circ2Traits® database was searched to confirm their relation to HCC.

*Methods*

*RNA extraction and reverse transcription*

Total RNA in patients and control samples was extracted using RNeasy® Mini Kit (50) Isolation System; (Qiagen, Valencia, CA, USA.) in keeping with the manufacturer’s guidelines (Cat. No. 217004). Nanodrop, POLARstar, Omega, and BMG Labtech was used to measure the intelligibility, pureness and concentration of the separated RNA. The cDNA was produced from extracted RNA by reverse transcription (RT) through a Revert Aid First Strand cDNA Synthesis; thermo scientific (Cat. No. K1622).

*Expression levels of hsa_circ_0064286 and hsa_circ_0000475 using Real-time polymerase chain reaction*

Real-time quantitative reverse transcription polymerase chain reaction (qRT-PCR) was achieved using SensiFASTTM SYBR No-ROX (Bioline) (Cat.NO BIO-98005), using a Real-Time PCR System; BioRad, miniopticon, USA. The used housekeeping gene (Reference gene) is Glyceraldehyde 3-phosphate dehydrogenase (GAPDH) (Lu et al., 2017).

The PCR primer sequences for both hsa_circ_0064286 and hsa_circ_0000475 were designed and manufactured by Sangon Biotech (Shanghai, China), while sequence of GAPDH was supplied by Qiagen, (Valencia, CA, and USA) as shown in (Table 1). 

- Calculation of Relative Quantification (RQ) (fold expression):

Cycle threshold (CT) values for hsa_circ_0064286, hsa_circ_0000475 and the house keeping gene (GAPDH) were determined. Expression levels changes of assessed genes relative to *GAPDH* gene were calculated using the ΔCt method (Schmittgen and Livak, 2008) according to the following equation: 

(1) Δ CT= CT measured gene – CT reference gene (*GAPDH*).

(2) ΔΔ CT = Δ CT HCC sample – Δ CT healthy (control) sample.

(3) RQ (Relative Quantification) = 2^– (ΔΔ CT)^

*Statistical analysis*

All statistics were performed and envisioned by Graph Pad Prism 7.0 (Graph Pad Software, La Jolla, CA, USA) and the Statistical Product and Service Solutions (SPSS) 22.0 software package (IBM, Chicago, IL, USA). All normally distributed data were expressed as mean ± standard deviation while the abnormally distributed data were expressed as median and interquartile range (IQR). Variances in the levels of expression of hsa_circ_0064286, 0000475 and liver biochemical markers were explored by ne-way analysis of variance (ANOVA) and independent sample t test. Possible correlations were measured by Pearson’s correlation test and spearman’s correlation tests. Chi-square test were used in demographic statistical analysis (age and gender). P value less than 0.05 was considered to be statistically significant. A receiver operating characteristic (ROC) curve was plotted from the results to determine the specificity and sensitivity of both circular RNAs. Circ RNAs expression levels were presented as relative expression and were analyzed through the Livak delta CT method (Schmittgen and Livak, 2008). All studied circular RNA data of different groups was compared to control group.

## Results

*The demographic and clinical characteristics data of the studied subjects*

A significant difference was noticed between HCC patients and the normal healthy group in the following parameters: liver biochemical function parameters, AFP, presence of HCV and of cirrhosis as shown in (Table 2).

*Expression levels of hsa_circ_0064286*

**Figure 1:**
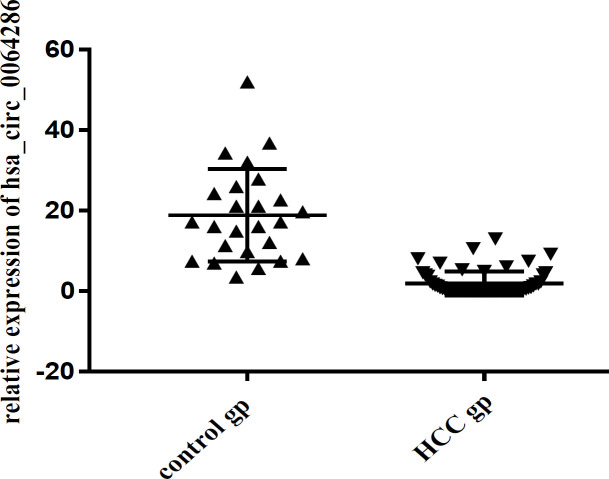
Hsa_circ_0064286 Expression Levels in all Studied Groups; p value <0.001; Abbreviations, Hsa, Homo sapiens; HCC, hepatocellular carcinoma

Serum hsa_circ_0064286 expression levels in HCC patients were significantly down regulated than those of control group with relative expression of (1.9±2) and (19±11), respectively, (P<0.001) as shown in (Figure1).

*Expression levels of hsa_circ_0000475*

**Figure 2:**
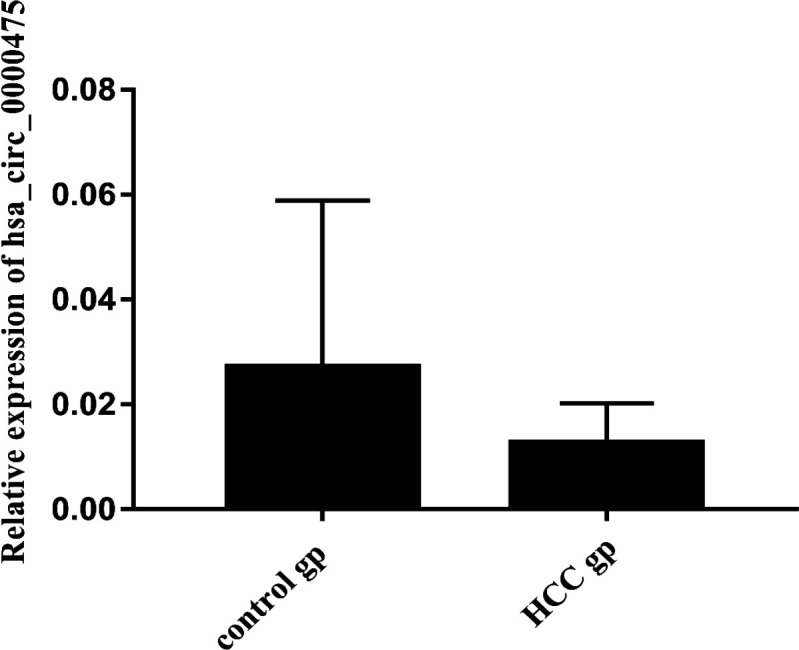
Hsa_circ_0000475 expression levels in all studied groups; p value <0.05; Abbreviations, Hsa, Homo sapiens; HCC, hepatocellular carcinoma

Results revealed that there was a significant reduction in serum hsa_circ_0000475 expression levels in HCC patients in comparison to control group with relative expression of 0.01 (0.003-0.02) and 0.03 (0.009-0.06), respectively, (P<0.05) as shown in (Figure 2).

*Association of hsa_circ_0064286 and hsa_circ_0000475 expression levels in HCC patients according to clinicopathological factors*

In our study, no significant difference was observed between hsa_circ_0064286 or hsa_circ_0000475 relative expression levels and any clinicopathological features of HCC patients such as child-Pugh classification, appearance of cirrhosis, tumor number, tumor size and HCV. 

*The Correlation between expression levels of hsa_circ_0064286 and hsa_circ_0000475 and liver biochemical parameters (* Table 3 *).*

There were positive correlations of both studied circular RNAs levels and serum albumin, on the other hand ALP, ALT, AST, AFP and bilirubin were negatively correlated with both studied circular RNAs as shown in (Table 3).

*Assessment of predictive performance of Circ_0000475 and Circ_0064286*

Receiver operating characteristics (ROC) curve was performed, hsa_Circ_0064286 area under the curve (AUC) was 0.971±0.015, with sensitivity and specificity 88.3% and 96% respectively, while hsa_circ_0000475 AUC was 0.679±0.064, with sensitivity and specificity of 78.3% and 56% respectively. 

## Discussion

Serum circRNAs levels were correlated with the pathogenicity of numerous diseases in human, including cancer (Holdt et al., 2016; Liu et al., 2016; Zhao et al., 2016; Zhao et al., 2017; Bai et al., 2018; Geng et al., 2018). CircRNAs are known to have interpretative and distinctive roles in cancer development and metastasis (Guarnerio et al., 2016; Liang et al., 2017).

CircRNA is believed to be responsible for RNA interlink network, the structure of circular RNAs are extremely abundant, relatively stable, diverse and conserved (Jeck et al., 2013).

Some CircRNAs have been reported in HCC either by increasing or decreasing in their expression levels, the expression levels of both hsa_circ_0005986 (Fu et al., 2017) and circMTO1 (Han et al., 2017) are decreased in serum of hepatocellular carcinoma patients while hsa_circ_0000673 expression levels was up regulated in HCC (Jiang et al., 2018). In the current study, circRNAs hsa_circ_0064286 and circRNAs hsa_circ_0000475 expression levels were lower in the sera of hepatocellular carcinoma patients compared to the healthy control. 

The observed lower levels were correlated with the levels of liver biochemical parameters (ALP, ALT, AST, AFP, bilirubin and albumin). It is well established that measurements of serum levels of liver functional parameters reflect the pathological state of liver and are used to monitor the prognosis of liver disease (Hayes and Chayama, 2016). Therefore, the expression levels of both circular RNAs hsa_circ_0000475 and circular RNAs hsa_circ_0064286 reflected the HCC aggressiveness in the studied patients.

It was proved that circRNAs have central role in regulating the gene expression through binding to sponge miRNAs (Thomas and Sætrom, 2014; Zhong et al., 2018) such as CircMTO1 which inhibits HCC proliferation and tumor growth through sponging miR-9 and p21 up-regulation (Han et al., 2017) and hsa_circ_0000673 whose oncogenicity stemmed from sponging of miR-767-3p (Jiang et al., 2018). 

In the current study Circinteractome database was used to identify the possible miRNA targets and RNA binding protein for both hsa_circ_0064286 and hsa_circ_0000475. Only miRNAs speculated for hsa_circ_0064286 is miR-421 which was remarkable for the high complementarity in binding sites of hsa_circ_0064286, suggesting that miR-421 may be a target for hsa_circ_0064286. MiRNA 421 was reported to suppress a serine/threonine kinase, MAPK14, which regulates apoptosis and cell cycle, ultimately suppressing cellular proliferation and tumor formation (Hui et al., 2007; Thornton and Rincon, 2009). Since, the gene expression level of *MAPK14* might be suppressed by miR-421 (Xu et al., 2019), it was suggested that these effects may be reversed by hsa_circ_0064286. We suggested that hsa_circ_0064286 may inhibit tumorigenesis of HCC through the hsa_circ_0064286/miR-421/ MAPK14 axis and may inhibit miR-421-mediated MAPK14 degradation through competitive endogenous RNA mechanism. 

Also, upon screening CircInteractom database, we identified miR-665 that could bind tightly with hsa_circ_0000475. It was shown that miR-665 is overexpressed in hepatocellular carcinoma tissues (Hu et al., 2018). The tumor suppressor tyrosine phosphatase receptor type (PTPRB) is the target gene of *miR-665 *(Hu et al., 2018). PTPRB can be inactivated in human cancers through genetic mutations (Wang et al., 2004; Julien et al., 2011). So the up regulation of MiR-665 leads to inhibition of tumor suppressors *PTPRB* gene and since the expression of PTPR is regulated by miR-665 (Hu et al., 2018), therefore we assumed that, our studied hsa_circ_0000475 down-regulation could play a role in tumorigenesis of HCC. 

By using Circinteractome data base, the current study focused on identifying potential RNA-binding proteins (RBPs) of hsa_circ_0064286 according to the assumption that circRNAs may sponge protein (Guo et al., 2014; Qu et al., 2015). Eukaryotic initiation factor 4A-III (EIF4AIII) has 26 protein binding sites in hsa_circ_0064286. EIF4AIII is involved in regulating of different metabolic processes by RNA or other nucleic acids interactions (Mohamadkhani, 2014). 

It was found that HCC cellular proliferation and migration could be suppressed through knocking down EIF4AIII, which expression level increased in HCC (Zhang et al., 2020). Thus, it might be suggested that EIF4AIII may bind to hsa_circ_0064286 causing its downregulation and inhibition of its possible tumor suppressor activity. 

However, detailed molecular mechanisms of hsa_circ_0064286 and hsa_circ_0000475 in liver cancer need to be more revealed.

Finally, sensitivity and specificity of both studied CircRNAS was calculated from ROC curve to inspect the possibility of applying either hsa_circ_0064286 or hsa_circ_0000475 as a diagnostic biomarker. It was found that hsa_circ_0064286 is more sensitive (88.3%) and specific (96%) than hsa_circ_0000475 whose sensitivity and specificity were 78.3% and 56%, respectively. As far as we know, the current study is the first one that detect the expression levels of hsa_circ_0064286 and hsa_circ_0000475 in the sera of HCC patients and their correlation with liver parameters.

In conclusion, circular RNAs hsa_circ_0064286 and hsa_circ_0000475 expression was down regulated in HCC patients; thus it is possible that they might serve as novel potential biomarkers that contribute to HCC diagnosis. Moreover, there is a possibility to direct the research towards both circRNAs with specific attention to hsa_circ_0064286 as therapeutic targets of novel approaches for treatment of HCC through targeting their sponge miRNAs or related proteins.

**Table 1:** Sequences of qRT-PCR Primers

Column1	Forward	Reverse
Hsa_circ_0064286	5′-TGTGGCATCTGCTGACCTCCTG-3′	5′-AGGCACTACTAAAAGGGGCAAACTG-3
Hsa_circ_0000475	5′-ACCAAAGTCGGCACATTCTCTTACG-3′	5′-CGCTGATGAAGTGGGGTGAACAC-3′
GAPDH	5′- CGGAGTCAACGGATTTGGTCGTAT -3′	5′- AGCCTTCTCCATGGTGGTGAAGAC -3′

Notes, GAPDH as housekeeping gene; Abbreviations, GAPDH, glyceraldhyde 3 phosphate dehydrogenase; Hsa, Homo sapiens.

**Table 2:** Demographic and Clinical Characteristics Data of All the Studied Subjects

Parameters		HCC N=60		Control N=25	
		n	%	n	%
Gender	Female	25	42%	12	48%
	Male	35	58%	13	52%
Age	<60	34	57%	13	52%
	≥60	26	43%	12	48%
ALT (U/l)	≤50	32	53%	25	100%
	>50	28	47%		
AST (U/l)	≤45	13	22%	25	100%
	>45	47	78%		
AFP (ng/ml)	≤40	7	12%	25	100%
	>40	53	88%		
HCV	Positive	49	82%		
	Negative	11	18%	25	100%
Cirrhosis	Present	41	68%		
	Absent	19	32%	25	100%

Abbreviations, AFP, alpha feto protein; ALT, alanine transaminase; AST, Aspartate transaminase; HCV, hepatitis C virus; Notes: n, case number; %, percent; total number of HCC, 60; total number of control, 25.

**Table 3:** The Correlation between hsa_circ_0064286 and hsa_circ_0000475 Expression Levels with the Liver Biochemical Parameters

Markers	Hsa_circ_0064286		Hsa_circ_00004754	
Liver biochemical parameters	Correlation (r)	P value	Correlation (r)	P value
ALP	-0.340	P<0.01	-0.246*	P<0.05
AST	-0.307	P<0.01	-0.228*	P<0.05
ALT	-0.309	P<0.01	-0.229*	P<0.05
AFP	-0.307**	P<0.01	-0.243*	P<0.05
Albumin	0.702**	P<0.01	0.218*	P<0.05
Bilirubin	-0.214*	P<0.05	-0.279**	P<0.01

Abbreviation: AFP, alpha feto protein; ALP, alkaline phosphatase; ALT, alanine transaminase; AST, Aspartate transaminase; Notes, *, P<0.05; **, P<0.01

## Author Contribution Statement

Fathia Zaky El Sharkawi: Constructed the idea and the design of the experiment, supervising the practical work , participated in the analysis of the data and interpretation of results, reviewed the manuscript. M. Samy Awad: Collected the patient’s samples, carried out the practical work and statistical analysis, participated in writing the manuscript. Waleed Elagawy: Responsible for the clinical data of the patients and aids in blood sample collection. H.A. Al-Sawaf: Participated in the revision of results and statistical analysis. Heba Taha: participated in the design of the work, participated in the analysis of the data and interpretation of results, preparing the final form of manuscript for submission.

## Part of student thesis

This research is a part of student Master’s thesis.

## Ethical committee approval

The Human Research Ethics Committee of Helwan University approved all aspects of this study with approval number (04H2020).

## Availability of data and material

The clinical and demographic data of all patients are available upon request.

## Registration of dataset

This research is not registered in any clinical trials, guideline or meta-analysis.

## Conflict of Interest/Competing Interests

The authors report no conflict of interest including any financial, personal or other relationships with other people or organizations that could inappropriately influence, or be perceived to influence the work in this paper.
